# Rubeosis iridis

**DOI:** 10.11604/pamj.2017.28.279.13717

**Published:** 2017-11-29

**Authors:** Dionysios Pagoulatos, Constantine Georgakopoulos

**Affiliations:** 1 Department of Ophthalmology, Hippokration Hospital, Athens, Greece; 2 Department of Ophthalmology, Medical School, University of Patras, Patras, Greece

**Keywords:** Neovascularization, diabetic retinopathy, neovascular glaucoma

## Image in medicine

A 49 year-old man with medical history of diabetes type 2, who presented to the ophthalmology clinic for decreased vision. His most recent hemoglobin A1c was 11.4%. His intraocular pressures were 19 mmHg OD and 35 mmHg OS. Posterior segment exam showed severe proliferative diabetic retinopathy in both eyes. Neovascularization of the iris (NVI), also known as rubeosis iridis, is when, blood vessels develop on the anterior surface of the iris in response to retinal ischemia. This condition is often associated with diabetes in advanced proliferative diabetic retinopathy, central retinal vein occlusion, ocular ischemic syndrome and chronic retinal detachment. These new blood vessels may cover the trabecular meshwork and give rise to neovascular glaucoma. Patients with NVI are prone to spontaneous hyphemas as these blood vessels are fragile. Patients with proliferative diabetic retinopathy who develop NVI are treated with panretinal photocoagulation with or without an intravitreal injection of an anti-VEGF medication and with glaucoma treatment.

**Figure 1:**
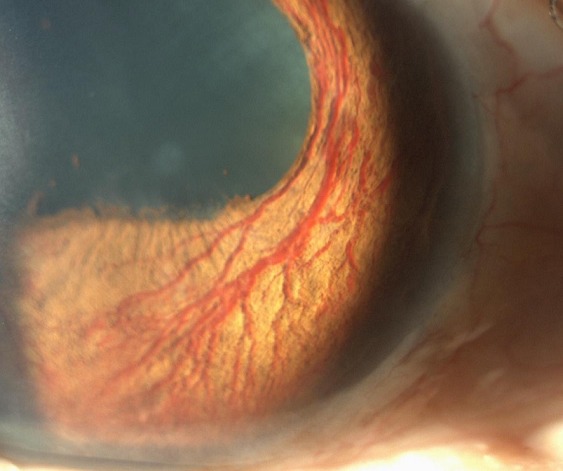
Rubeosis iridis

